# Oral corticosteroid use for clinical and cost-effective symptom relief of sore throat: study protocol for a randomized controlled trial

**DOI:** 10.1186/1745-6215-15-365

**Published:** 2014-09-18

**Authors:** Johanna Cook, Gail Hayward, Matthew Thompson, Alastair D Hay, Michael Moore, Paul Little, Kim Harman, Jane Wolstenholme, Rafael Perera, Merryn Voysey, Julie Allen, Maria Breen, Carl Heneghan

**Affiliations:** Nuffield Department of Primary Care Health Sciences, University of Oxford, Radcliffe Observatory Quarter, Woodstock Road, Oxford, OX2 6GG UK; Centre for Academic Primary Care, NIHR School for Primary Care Research, School of Social and Community Medicine, University of Bristol, Canynge Hall, 39 Whatley Road, Bristol, BS8 2PS UK; Aldermoor Health Centre, Aldermoor Close, Southampton, SO16 5ST UK; Department of Family Medicine, University of Washington, 1959 Ne Pacific St, Seattle, WA 98195 USA

**Keywords:** Sore Throat, Steroid, Antibiotics, Delayed Antibiotic Prescription, Dexamethasone, Same Day Recruitment, Adults, Randomized, Placebo-controlled

## Abstract

**Background:**

Management of acute sore throat poses a significant burden on UK general practices, with almost 10% of registered patients attending their GP with sore throat every year. Nearly half of all patients presenting with acute sore throat are treated with antibiotics, despite their limited effect. In a recent systematic review we demonstrated that a single dose of steroids reduced the severity and time to resolution of sore throat. However, all of the trials included looked at the use of steroids alongside antibiotics and only one was in a primary care setting. This trial aims to assess the efficacy and cost-effectiveness of a single oral dose of corticosteroids on symptoms of sore throat in patients receiving either a delayed antibiotic prescription or no antibiotics at all in UK primary care.

**Methods/Design:**

A double-blind, two arm, randomized, placebo controlled trial in adults (≥18 years of age) presenting to primary care with acute sore throat (<seven days). Participants are recruited on the day of presentation to their GP practice. GPs or nurses assess eligibility, record baseline clinical features and obtain a throat swab for bacterial culture. Participants are being randomized to treatment arms at a ratio of 1:1. Treatment arms will be stratified according to whether patients are being given a delayed antibiotic prescription or no antibiotic prescription and by recruiting centre (Oxford, Bristol or Southampton). Outcome data is being collected at 24 and 48 hours via text message or telephone call, from days 0 to 7 using a patient symptom diary and at one month via a GP notes review.

**Discussion:**

This will be the first randomized controlled trial of oral corticosteroids in adults presenting to primary care with sore throat in the UK, and the first to examine the clinical and cost-effectiveness of oral corticosteroids for the treatment of sore throat in the absence of antibiotics.

**Trial registration:**

This trial is registered with Current Controlled Trials on 26 March 2013, registration number: ISRCTN17435450.

**Electronic supplementary material:**

The online version of this article (doi:10.1186/1745-6215-15-365) contains supplementary material, which is available to authorized users.

## Background

### Epidemiology, costs and current management of sore throat

Sore throat is a common complaint and is a frequent reason for patients to attend primary care. In 2006, nine patients consulted a general practitioner (GP) with sore throat for every 100 patients registered [1]. Tonsillitis was diagnosed in 3 out of 100 patients registered, and of these, 91% received antibiotics. Half of the remaining cases, coded as sore throat or pharyngitis, also received antibiotics. Prescribing rates for sore throat are clearly disproportionately high, especially since treatment of sore throat with antibiotics provides only modest symptomatic benefit [2, 3].

Antibiotic resistance in general is still increasing across Europe and represents a growing threat to the effectiveness of antibiotics [4–6]. Although prescribing rates have fallen for patients presenting with the common cold, a similar decrease has not been noted for sore throat [1]. Part of the reason may be the absence of alternative symptomatic treatments, resulting in a prescribing ‘vacuum’.

The lost productivity associated with tonsillitis has been estimated at £190 per episode [7]. The weekly UK incidence of patients presenting to their GP with sore throat averages at 60 per 100,000 in the current population. Extrapolating from this, we might expect a cost of almost £6 per person per year in lost productivity alone (equating to £370 million at 2010 population figures), in addition to an estimated £60 million cost in GP consultations [8].

### Rationale for testing the effectiveness of corticosteroids in sore throat

Corticosteroids may offer an alternative symptomatic treatment for sore throat. They are known to inhibit transcription of the pro-inflammatory mediators in airway endothelial cells which are responsible for pharyngeal inflammation and ultimately symptoms of pain [9]. Steroids are beneficial in other upper respiratory tract infections such as acute sinusitis, croup, and infectious mononucleosis [10–13]. Short courses of even high-dose oral steroids are considered to be safe, in the absence of any specific contraindications [14].

A systematic review and meta-analysis of randomized controlled trials assessing the benefit of oral corticosteroids in sore throat [15, 16] identified eight eligible trials. The review found that a single dose of oral or intramuscular dexamethasone increased the likelihood of complete resolution of pain at 24 hours by more than three times (relative risk 3.2 (95% CI 2.0 to 5.1; *P* <0.001), absolute risk reduction 27% (95% CI 17 to 36%), number needed to treat 3.7 (95% CI 2.8 to 5.9)). The mean time to onset of pain relief was reduced by more than 6 hours (95% CI 3.4 to 9.3; *P* <0.001). However, all of the included trials compared steroids to placebo in addition to oral antibiotics. Furthermore, only one of the trials (in Israel) recruited patients presenting to primary care. We have searched the International Controlled Trials Register to confirm there are no similar trials currently being conducted or registered.

### Justification for treatment dose and method

The dose of oral corticosteroid used in the majority of previous trials in adults was a single dose of 10 mg of dexamethasone or the equivalent dose of prednisolone, either orally, or intramuscularly, or both. Those trials included children up to the age of 18-years-old and used 10 mg of dexamethasone as the maximum dose. Our systematic review found no difference in the effect of oral compared to intramuscular administration of corticosteroid. Therefore this trial is using a single dose of 10 mg of oral dexamethasone as the dose most commonly found to be effective and the treatment method causing the least discomfort.

### Known and potential risks to human participants

Long-term steroid use is known to be associated with an array of systemic side effects [17]. However, in the absence of specific contraindications [17, 18], a short (up to 1 week) course of high-dose steroids is considered to be safe and associated with few side effects [19]. Our systematic review found no difference in either serious adverse events or all adverse events, relapse or recurrence rates between participants receiving corticosteroids and those receiving placebo [15].

The prospect of achieving rapid symptomatic relief with a single dose of oral steroids has positive implications: improving patient treatment options, reducing unnecessary antibiotic prescriptions and reducing the economic burden of sore throat. However, evidence is required for the clinical and cost-effectiveness of oral steroids in sore throat in the absence of antibiotics and in a primary care population. We are therefore conducting a randomized double-blind trial comparing a single dose of oral dexamethasone to placebo in adults aged 18 years or over presenting to UK primary care (Treatment Options without Antibiotics for Sore Throat or TOAST trial).

## Methods/Design

### Objectives

The primary objective of the trial is to investigate if, in adults ≥18 years presenting to primary care with acute sore throat, the use of a single dose of oral dexamethasone leads to increased speed of resolution or improvement in symptoms compared with placebo.

The trial has several secondary objectives. Firstly, to investigate whether dexamethasone compared with placebo leads to increased resolution or improvement in symptoms in those patients who have not been prescribed antibiotics. Secondly, to investigate whether dexamethasone compared to placebo will, in those patients offered a delayed antibiotic prescription, reduce the number of patients taking antibiotics for their sore throat within seven days. Thirdly, to investigate whether a single dose of oral dexamethasone compared to placebo will: reduce time away from work or education within seven days, not increase the incidence of hospital admission with complications related to sore throat (such as peritonsillar abscess) within 28 days, not increase repeat attendance at the GP within 28 days with symptoms or complications of sore throat, and be cost-effective. Fourthly, to assess predictors of response to corticosteroids including existing severity scores (FeverPAIN score and Centor score), baseline factors and positive bacterial throat swab.

### Trial design

The trial is a two arm, individually randomized, double-blind trial comparing a single dose of 10 mg oral dexamethasone with placebo in adults aged 18 years or over presenting to primary care with sore throat. The trial requires a single visit to the GP from each participant and a one-week period of participant involvement from the point of randomization and treatment. See flow chart (Additional file 1). The trial will be a multicenter trial based at general practices in Oxford, Bristol and Southampton in England, UK.

### Primary and secondary endpoints and outcome measures

#### Primary outcome

The primary outcome of this trial is the direct report by the patient of presence or absence of complete resolution of sore throat at 24 hours by either text message or telephone.

#### Secondary outcomes

The trial has several secondary outcomes which include: direct report by those patients who have not been prescribed antibiotics of presence or absence of complete resolution of sore throat at 24 hours by either text message or telephone, and report of presence or absence of complete resolution of sore throat at 48 hours by either text message or telephone contact. Additionally we will record for the seven days after treatment is administered: time to onset of pain relief (in hours), time to complete symptom resolution (in hours), report of difficulty swallowing and pain on swallowing, the duration of moderately bad symptoms recorded by validated symptom diary, the change in ratings of sore throat pain and pain on swallowing by visual analogue scale, the uptake of delayed antibiotic prescription and any time missed from work or education.

Furthermore we will record the severity of symptoms in the two to four days after seeing a GP based on the symptom diary, any attendance at GP practices, Emergency Departments, or out-of-hours centers within 28 days with symptoms or complications associated with sore throat such as peritonsillar abscess, any hospital admission with related complications of sore throat within 28 days, use of over-the-counter medications and prescription medications (including, if delayed antibiotics are taken, whether the course is completed and whether any other antibiotics were taken) in the first seven days and cost-effectiveness measures which include incremental cost, EuroQol EQ-5D-5L score change in seven days and impact on usual activities over most recent seven days

### Inclusion criteria

Trial participants will be anyone aged 18 years or over presenting to primary care with acute sore throat which is judged by the clinician to be infective in origin, whose onset of symptoms has been within seven days of presentation and has the capacity and willingness to complete trial documentation.

### Exclusion criteria

The participant may not enter the study if any of the following apply:

They are a female participant who is pregnant, lactating or planning pregnancy during the course of the study, they have had recent (<one month) use of inhaled or oral corticosteroids or a recent (<one month) adenotonsillectomy.

Participants will be excluded if they are currently or recently (<14 days) taking antibiotics, there is a clear alternative diagnosis such as pneumonia or they have a known immune deficiency (such as HIV, active chemotherapy or advanced cancer), are scheduled elective surgery or other procedures requiring general anaesthesia during the next seven days, are terminally ill, or have symptoms or signs suggesting that hospital admission is required (such as being completely unable to swallow, very systemically unwell or peritonsillar abscess).

They are also excluded if the patient is judged by the GP to require immediate antibiotics, has a history of severe affective disorders including steroid-induced psychiatric illness, is currently taking any medication on the British National Formulary (BNF) listed contraindications to oral steroids or has existing symptoms that are also side effects of oral steroids or if the patient is taking other interacting medication (for example phenytoin and anti-coagulants). Clinicians are asked to use the BNF and their clinical prescribing systems to check for interactions for all patients.

Further to this patients are excluded if they have a known dexamethasone allergy or if they have any other significant disease or disorder which, in the opinion of the investigator, may either put the participants at risk because of participation in the study, or may influence the result of the study or the participant’s ability to participate in the study.

If the patient has been involved in another clinical trial of an investigational medicinal product in the last 90 days or any other research within the last 30 days, the recruiting primary care site is not the patients usual practice if the patient is not expecting to still be with the primary care site in one month ( temporary residents), they have previously participated in the TOAST trial the patient is unable to be randomized by the end of the (working) day of presentation or they have a requirement for a live vaccine in the next seven days they will also be excluded.

### Expenses and benefits

Healthcare visits in addition to normal care are not anticipated, nor does the trial offer any other payment for involvement in the study, however if additional travel expenses are incurred due to an extra visit to the GP for the baseline trial assessment these expenses will be covered. Trial medication is provided free of charge, but participants will have to pay for their antibiotic prescription, if one is given. However, participants who return a completed symptom diary to the Primary Care Clinical Trials Unit (PC-CTU) are being sent a £10 gift card as a thank you for participating and completing all the follow-up documentation.

### Study procedures

#### Informed consent

The participant must personally sign and date the latest approved version of the informed consent form before any study specific procedures are performed. Written and verbal versions of the participant information sheet and informed consent are presented to participants by the responsible clinician detailing no less than the exact nature of the study, the implications and constraints of the protocol, the known side effects and any risks involved in participation. It is clearly stated that the participant is free to withdraw from the study at any time for any reason without prejudice to future care, and with no obligation to give the reason for withdrawal.

The participant receives the participant information sheet at their initial consultation with their GP, and if eligible and interested, is referred on to a baseline trial assessment with a recruiting clinician for full consent procedures and trial procedures. This gives participants the opportunity to consider the information, and to question the recruiting clinician, their GP or other independent parties to decide whether they will participate in the study. Written informed consent is then obtained by means of participant dated signature and dated signature of the person who presented and obtained the informed consent. The person who obtained the consent must be suitably qualified, experienced and trained in line with the Medicines and Healthcare Products Regulatory Agency, MHRA requirements, and have been authorized to do so by the Chief Investigator. A copy of the signed informed consent will be given to the participants and the original signed form will be retained at the study site.

#### Screening and eligibility assessment

The primary care site gives adults presenting with sore throat a participant information sheet which details what is involved in trial participation. During the initial consultation the primary care clinician (referred to from now onwards as the responsible clinician) discusses trial participation and screens the participant using the inclusion and exclusion criteria. The responsible clinician may be a triage nurse if the GP judges that they are competent to perform the baseline assessment and eligibility screening. Any patient who is not eligible to participate or declines to participate will be recorded on the screening log with reasons for ineligibility or declining (if known) and have no further involvement in the trial.

The responsible clinician completes their routine clinical management, and at the clinician’s discretion will offer a delayed antibiotic prescription, to be collected by the patient either from the recruiting clinician at their subsequent baseline trial assessment, or from the reception of the GP practice according to the standard procedure of the practice. The delayed antibiotic prescription will be accompanied by: reassurance that antibiotics are often not needed immediately and information about the disadvantages of antibiotics, information about the natural history of sore throat and advice to use regular pain relief, instructions for the antibiotics to be collected and used after three to five days if the patient feels their symptoms are not starting to improve, or sooner if their symptoms are getting significantly worse and a brief information leaflet containing instructions and explanation regarding a delayed prescription to reinforce these points.

#### Baseline assessments

Within six hours of the initial consultation, potentially eligible patients undergo a baseline trial assessment with a primary care clinician allocated by the practice to recruit patients (from here on known as the recruiting clinician).

At this meeting a full trial explanation is given and time allowed for the participant to ask any questions, and then written consent is obtained. The recruiting clinician uses a secure, web-based data collection platform (hosted by the University of Oxford) to enter the participant’s baseline data and confirm eligibility using a standard computer within the GP practice. Once the eligibility is confirmed, randomization proceeds.

The recruiting clinician gives the participant standardized instructions regarding how to complete the symptom diary and other response forms and observes the participant taking the trial medication (oral corticosteroid or placebo). The recruiting clinician records the participant’s contact details for the 24- and 48-hour data collection contacts. Those participants for whom the GP has deemed a delayed antibiotic prescription is appropriate are provided with the prescription if this is the standard procedure of the practice.

The recruiting clinician takes a bacterial throat swab. These are being analyzed for streptococcus A, C and G. The participant’s date of birth, sex and the participant trial ID number are being used as identifiers for these swabs. In the rare event that an unusual and potentially dangerous pathogen is detected by bacterial throat swab, and the medically qualified Chief Investigators feel it is appropriate, the practice will be informed of these results.

Baseline case report form data items are:

Socio-demographic factors which will include: age, gender, smoking history and paid work or education. Medications which will include: decision whether or not to offer delayed antibiotic script and if offered, type of dose, dosing regimen and duration of antibiotics prescribed as well as whether the practice left the script for collection at reception or gave it to the patient at the baseline recruitment meeting and any other advised treatment. Symptoms which will include: the duration of sore throat and painful swallowing and the presence or absence of cough, hoarse voice, coryza and fever in last 24 hours, all of which use a validated scale, where ‘None’ is equal to absence of and ‘Slight’ ‘Moderate’ and ‘Severe’ are equal to presence of. Clinical examination findings which will include the confirmation of: the presence of pharyngeal inflammation, the presence of tonsils, the presence of inflamed tonsils, the presence of purulent tonsils, the presence of cervical lymphadenopathy, the presence of tender cervical lymphadenopathy, the participants temperature and type of thermometer used for measuring. Patient-completed items which will include: ratings of throat soreness, pain on swallowing and difficulty swallowing using visual analogue scales, baseline severity ratings using symptom diary and completion of the EuroQol EQ-5D-5L instrument [20].

All these procedures happen according to the schedule of procedures; see Additional file 2.

#### Randomization and codebreaking

Randomization was performed by the Oxford PC-CTU and is stratified by centre (Oxford, Bristol and Southampton) and by receipt or not of delayed antibiotic prescription using a block randomization with variable block size. An independent statistician based in the Department of Primary Care Health Sciences at the University of Oxford generated the randomization schedule. They produced a list of four-digit unique medication IDs; these were printed on the medication labels in variable block sizes stratified as above. This statistician is not involved in any other aspect of the trial.

Each site is initially allocated to hold two sets of two to three packs of pre-randomized medication, one set for those who are given an antibiotic prescription and one set for those who are not. They then liaise with their local centre (the centre responsible for setting up the site) when they have allocated their existing packs to trial participants and reallocation of medication, if deemed necessary, only occurs within the same centre and same subgroup of participants, having delayed antibiotic prescription or not. They also receive an equal number of participant folders containing unique participant trial IDs.

The recruiting clinician allocates the patient one pack of medication from the appropriate set of pre-randomized medications and they record the unique medication ID on the baseline case report form, CRF. The recruiting clinician informs their local study centre (Oxford, Bristol or Southampton) which medication has been allocated to which participant trial ID and the local study centre keeps a log of all allocated medication and participant trial IDs. The recruiting clinician also enters the participant trial ID on the drug allocation log on site against the allocated medication ID.

The trial investigators have reviewed the clinical safety of the study and do not feel that an emergency 24-hour unblinding service is required. The only major adverse event where clinical management might be affected by this knowledge is anaphylaxis, and, as the medication is taken by the participant under observation in the general practice during working hours, this will be managed in hours if required. In addition an independent clinician has confirmed that clinical care offered to a patient presenting with an adverse event or serious adverse event, AE/SAEwould not be influenced by knowledge of which study arm they were in. Participants remain in the practice for 10 minutes after the medication has been taken to ensure that any immediate reaction can be treated. In the very rare event that analysis of the bacterial throat swab reveals an unusual and potentially dangerous pathogen; the Chief Investigator will be contacted to assess the need for emergency unblinding and informing the participant’s practice. This information will only be received, and the practice contactable, in office hours.

A standardized procedure for emergency unblinding is available. The codes will only be broken in case of a major adverse event (such as anaphylaxis or admission to hospital with a life-threatening illness (for example septicemia, meningitis, severe pneumonia requiring ITU admission or death)). The randomization code is stored electronically on a secure password-protected drive and access is restricted to the independent statistician. If unblinding is deemed necessary the Chief Investigator or designated representative will inform the independent statistician to notify the relevant responsible clinician of the treatment allocation for the relevant participant. The trial investigators will not be informed which arm of the trial the participant was allocated to. If randomization of a participant is unblinded during the study then data for that participant, if available, will be included in the intention-to-treat analysis.

The procedures for code break at the end of the trial will be as follows: once all the data queries have been resolved, a blind data review meeting will be initiated involving the trial statistician, the data manager, the trial manager and the Chief Investigator. All protocol violations will be reviewed and a list of study populations for analysis will be generated and signed off by the Chief Investigator and the statistician. At this point, the database will be locked and decoding of the allocation will be allowed.

#### Subsequent assessments

Following the baseline trial assessment participants complete a symptom diary reporting upon the resolution of symptoms and time to onset of pain relief, and rating their pain on a visual analogue scale every day for seven days on paper. As well as recording the severity and duration of their symptoms, this also includes providing information about NHS resource use, out-of-pocket expenditure, use of over-the-counter and prescription medications and time off work and/or education or foregone leisure time. Within the symptom diary we also ask participants to complete the EuroQol EQ-5D-5L instrument [20] daily for seven days following study entry.

Participants are telephoned or texted at 24 and 48 hours to support collection of the primary outcome and secondary outcomes. As preference this contact will be made via text message because of the greater accuracy with times and standardization. They are additionally telephoned in the first 96 hours if required to support and encourage completion of the symptom diary; they will also receive a text message at day four and day nine to encourage completion of the symptom diary and to remind them to return it to the PC-CTU. Follow-up is being undertaken by research coordinators at all three centers. Follow-up continues for seven days from the initial day of recruitment. Participants are asked to report in the diary any use of medications, including whether they obtained and completed the delayed antibiotic prescription. If participants do not complete the symptom diary over the seven days we send them a short questionnaire after this in order to collect information for key secondary outcomes relating to the first four days after taking the trial medication and, if needed, they are telephoned in order to help them complete the questionnaire. All paper diaries and questionnaires are sent back to the respective centre in pre-paid envelopes.

A review of the primary care notes is undertaken by the recruiting primary care site one month post-randomization, to record repeat presentation to a GP, Emergency Department or out–of-hours primary care centre with symptoms or complications of sore throat, hospital admissions and use of prescription medications. Baseline information about past medical history and acute and repeat medication usage is also collected.

### Definition of end of trial

The end of trial will occur once the primary outcome data has been collected for all patients and the one month follow-up notes review of the final participant has been performed.

### Discontinuation and withdrawal of participants from study treatment

Each participant has the right to withdraw from the study at any time. In addition, the investigator may discontinue a participant’s involvement in the study at any time if the investigator considers it would be harmful to keep a participant in the study. The reason for withdrawal will be recorded in the CRF.

If the participant is withdrawn due to an adverse event, the investigator will arrange for follow-up visits or telephone calls until the adverse event has resolved or stabilized, or until the end of the study when participant care will return solely to the GP. The participant’s data will be retained in the trial for the purpose of intention-to-treat analysis unless consent to do so is specifically withdrawn. This is in order to safeguard the validity of the data set. If a participant is found to be ineligible after they have been randomized then they will be removed from the trial. Their data will also be removed from the intention-to-treat analysis.

### Source data

Source documents are original documents, data and records from which participants’ CRF data are obtained. These include, but are not limited to, general practice medical records (from which medical history and previous and concurrent medication may be summarized into the CRF, as well as follow-up data at one month). CRF entries will be considered source data if the CRF is the site of the original recording (there is no other written or electronic record of data). In this study the CRF is being used as the source document for the documentation of inclusion and exclusion criteria, and baseline assessment information. Symptom diaries and telephone calls recorded onto CRFs are considered source data. All documents are stored safely in confidential conditions. On all study-specific documents other than the signed consent the participant will only be referred to by their study participant ID.

### Treatment medication

#### Description of study treatment

The study treatment is a single 10 mg dose of dexamethasone taken orally. The dose takes the form of five 2 mg dexamethasone tablets over-encapsulated into a single capsule and an over-encapsulated placebo identical in size, colour and taste. The drug acquisition, over-encapsulation, packaging and labelling was performed by Nottingham University Hospitals NHS Trust.

The labelling of medication packs conforms to Annexe 13 (GMP) and Article 13.3 of European Commission Directive 2001/20/EC. A template label was approved by the clinical trial team and provided to the manufacturer by the Chief Investigator. Each medication pack label is printed with a unique medication ID number to ensure dexamethasone and placebo medicine packs are indistinguishable and thus maintain allocation concealment. This randomized medication ID forms the identifier on the open code-break document sent with each delivery of medication packs to the University of Oxford PC-CTU. The medicines were received from the manufacturer and are stored securely by the clinical trials unit.

The trial centers are responsible for supplying the medication packs to the GP practices in their area, four to six packs at any one time, so that clinicians can draw from their allocation as recruitment proceeds. Trial centers keep a log of medication packs sent to a GP practice, with all medication packs signed for on receipt at the GP practice. Sites liaise with their local centre when more packs are required, and the local centre then liaises with the Oxford centre to send a further block to the local centre. At all times the medicines will be stored at room temperature, in line with the summary of product characteristics, SmPC. The study drug and placebo can be stored below 25°C and out of direct sunlight and are kept securely in the Oxford PC-CTU and on site, with controlled access by site trial staff. A formal risk assessment considering all the potential risks involved in the distribution of the trial medication will be written alongside a standard operating procedure detailing the exact procedure for the handling of all trial medication to ensure that any associated risks will be minimized.

#### Compliance with study treatment

The participant is observed taking the single dose of study medication once they have provided full informed consent.

#### Accountability of the study treatment

The study medication is supplied by Nottingham University Hospitals NHS Trust to the clinical trials unit. All movements of study medication between Nottingham University Hospitals NHS Trust and clinical trials unit are documented. The clinical trials unit will supply the allocated drugs to the local centers who distributed these out to the sites in their area. The clinical trials unit will keep logs of all medication IDs and where each drug is sent to, local centers will keep logs of all drugs allocated to them and the GP practices will keep local drug accountability logs, including drug allocation logs.

In the event that medication needs to be redistributed, a drug redistribution log must be completed to document the unique medication ID and must include a minimum of one release signature (origin site staff), one transporter signature (PC-CTU staff) and one receiving signature (new site staff), with approval from the MHRA.

Site-specific procedures will be followed in relation to disposing of and arranging for destruction of expired trial medication. Standard GP site procedures should be followed and the drug destruction log should be completed with the following details: date, unique medication ID, expiry date, quantity to be destroyed (number of tablets) and staff initials to confirm destruction.

#### Concomitant medication

Throughout the study the responsible clinician may prescribe any concomitant medications or treatments deemed necessary to provide adequate supportive care except for those listed in the exclusion criteria. If these are required, the participant will stay in the trial for purposes of intention-to-treat analysis. Any medication taken during the study other than the study medication will be recorded in the symptom diary or noted on notes review.

#### Post-trial treatment

Following the single dose of oral dexamethasone participants continue normal medical care by their GP.

### Safety reporting

#### Definitions

An adverse event (AE) or adverse experience is any untoward medical occurrence in a patient or any abnormal result from a clinical investigation in participants who have been administered a medicinal product,which does not necessarily have to have a causal relationship with this treatment (the study medication). An AE can therefore be any unfavorable and unintended sign (including an abnormal laboratory finding), symptom or disease temporally associated with the use of the study medication, whether or not considered to be related to the study medication. An adverse reaction (AR) will be defined as all untoward and unintended responses to a medicinal product related to any dose. The phrase ‘responses to a medicinal product’ means that a causal relationship between a study medication and an AE is at least a reasonable possibility - the relationship cannot be ruled out. All cases judged by either the reporting medically qualified professional or the Sponsor as having a reasonable suspected causal relationship to the study medication qualify as adverse reactions. A serious adverse event (SAE) will be defined as any untoward medical occurrence that at any dose results in death, is life-threatening, requires inpatient hospitalization or prolongation of existing hospitalization, results in persistent or significant disability and/or incapacity, is a congenital anomaly or birth defect or other important medical events. Other events that may not result in death, are not life threatening, or do not require hospitalization, may be considered an SAE when, based upon appropriate medical judgment, the event may jeopardize the patient and may require medical or surgical intervention to prevent one of the outcomes listed above. The term life-threatening in the definition of SAE refers to an event in which the participant was at risk of death at the time of the event; it does not refer to an event which hypothetically might have caused death if it were more severe.

To ensure no confusion or misunderstanding of the difference between the terms serious and severe, which are not synonymous, the following note of clarification is provided. The term severe is often used to describe the intensity (severity) of a specific event (as in mild, moderate or severe myocardial infarction). The event itself, however, may be of relatively minor medical significance (such as severe headache). This is not the same as ‘serious’, which is based on participant and/or event outcome or action criteria usually associated with events that pose a threat to a participant's life or functioning as defined in the bullet points above. Seriousness (not severity) serves as a guide for defining regulatory reporting obligations.

A serious adverse reaction (SAR) is defined as an AE (expected or unexpected) that is both serious and, in the opinion of the reporting investigator, believed with reasonable probability, to be due to one of the study treatments, based on the information provided. A suspected unexpected serious adverse reaction (SUSAR) is defined as an SAR, the nature or severity of which is not consistent with the applicable product information (such as an investigator’s brochure for an unapproved investigational product or summary of product characteristics for an approved product).

### Causality

The relationship of each AE to the trial medication must be determined by a medically qualified individual to be either related or not related. Related, for these purposes, requires the AE to follow a reasonable temporal sequence from trial medication administration. It cannot reasonably be attributed to any other cause. Not related, for these purposes, requires that the AE is probably produced by the participant’s clinical state or by other modes of therapy administered to the participant.

#### Recording and reporting procedures for AEs and SAEs

Dexamethasone is a commonly used medication in a primary care setting; it has well defined safety profiles and is being used in this trial for authorized indications. As a result of this no non-SAEs will be recorded in this study. All SAEs occurring during the one month participants are enrolled on the trial will be recorded.

A participant may voluntarily withdraw from the trial due to what he or she perceives as an intolerable AE. AEs that result in a participant’s withdrawal from the study will be recorded on the withdrawal form. The relationship of AEs to the study medication will be assessed by a medically qualified investigator. The severity of events will be assessed on the following scale: 1 = mild, 2 = moderate or 3 = severe.

All SAEs must be reported to the PC-CTU within one working day of discovery or notification of the event. The PC-CTU will perform an initial check of the report, request any additional information and ensure it is reviewed by the Chief Investigator on a weekly basis. The PC-CTU will also ensure that it is reviewed at the next Data Monitoring Committee meeting. All SAE information must be recorded on an SAE forms and faxed to the PC-CTU. Additional information received for a case (follow-up or corrections to the original case) need to be detailed on a new SAE form and faxed to the PC-CTU.

#### Data Monitoring Committee

The appointed and independent Data Monitoring Committee (DMC) conducts a review of all SAEs for the study reported during the quarter and cumulatively. They report their findings to the Trial Steering Committee who in turn report to the Trial Management Group. The main aims of this review are as follows: to ensure the safety and rights of each patient in the trial, to pick up any trends, such as increases in expected and unexpected events, and take appropriate action, to monitor the trial data and review and analyze as outlined in the statistical analysis plan, systematically or as requested by the Trial Steering Committee, to seek additional advice or information from investigators where required, to evaluate the risk, in terms of safety and ethics, of the trial continuing and take appropriate action where necessary and to act or advise, through the Chairman or other consultant, on incidents occurring between meetings that require rapid assessment. The Data Monitoring Committee will also suggest provision of training specific groups to the Trial Steering Committee as required.

#### SUSAR reporting

In collaboration with the University of Oxford’s Clinical Trials Research Governance (CTRG) the Chief Investigator will report all SUSARs to the competent authorities (MHRA in the UK), the research ethics committee concerned and host NHS Trusts. Fatal or life-threatening SUSARs will be reported within seven days and all other SUSARs within 15 days. Any additional relevant information will be reported within eight days of the initial report. The Chief Investigator will also inform all investigators concerned of relevant information about SUSARs that could adversely affect the safety of participants.

#### Development safety update reports

In addition to the expedited reporting above, the Chief Investigator shall submit once a year throughout the clinical trial on the anniversary of the clinical trial authorization CTAor on request a development update safety report (DSUR) to the competent authorities (MHRA in the UK), research ethics committee concerned, host NHS Trust and Sponsor.

### Statistics

#### Statistical analysis for effectiveness and safety

The primary analysis will be intention-to-treat assuming no resolution for missing data. The proportion of complete resolution at 24 hours reported by participants will be compared between two treatment arms using a generalized linear regression model for binary data adjusting for whether participants are prescribed antibiotics or not. The proportion of complete resolution at 24 hours in those participants who have not been prescribed antibiotics (on which this trial is powered) will be compared in the same way.

Logistic regression adjusting for whether participants are prescribed antibiotics or not will also be performed to estimate the differences in the proportions of binary secondary outcomes including reported complete resolution at 48 hours, hospital admission within 28 days, attendance at GP practice, Emergency Departments, or out-of-hours centers within 28 days with symptoms or complications associated with sore throat and uptake of delayed antibiotic prescription within seven days. We will explore whether positive bacterial throat swab, FeverPAIN score, Centor score and other baseline factors predict response to corticosteroid. Use of over-the-counter and prescribed medicine other than antibiotics will be summarized and compared using chi-square tests.

Mean and standard deviations for reported time to onset of pain relief, time to complete resolution of pain, duration of moderately bad symptoms recorded by validated symptom diary and time missed from work or education over the seven days from treatment onset will be calculated and compared between two treatment arms using a linear regression adjusting for antibiotic prescription. We will use data from participants’ diary on throat pain, pain on swallowing and difficulty in swallowing by visual analogue scale within seven days post-randomization to calculate areas under the curves as proxies for a summary measurement and tested for a difference between two arms using a linear regression adjusted for antibiotics prescription. All model assumptions will be assessed and if data do not conform to assumptions the alternatives will be explored. Symptoms of interest will be summarized in the proportions and difference between two treatment arms and 95% CI will be calculated. Full description of the methods to be used will be stated in a trial statistical analysis plan.

#### Health economics analysis

The objective of the economic evaluation is to establish the difference in costs associated with administering oral corticosteroids versus placebo for sore throat, and relate this cost differential to any difference in health benefits found. The economic evaluation will be undertaken alongside the trial using widely accepted methods and will take an NHS perspective. An evaluation from a wider societal perspective will also be undertaken (as a component of the cost-consequences analysis) as productivity losses and absenteeism are likely to be associated with sore throat. The costing exercise will identify the NHS services used.

The economic evaluation has been designed as a cost-utility analysis, using the participant’s EQ-5D-5L scores (using a published UK population valuation set and EuroQoL crosswalk algorithm [21]) as the main economic outcome measure. However, the performance and sensitivity of the EQ-5D-5Lin this participant group and over such a short follow-up period is uncertain, so its appropriateness will be investigated by assessing its construct validity and sensitivity to change within the trial. Due to the likely limitations in using EQ-5D-5L as the outcome measure, the cost-utility analysis will be supplemented by a cost-consequences analysis using a number of outcome measures (such as symptomatic days avoided, EQ-5D-5L disaggregated by domain and days off work and/or education) as the measure of health benefit.

Individual-level resource use data is being collected using resource-use questionnaires and GP records. The resource use data covers general practice, medications and hospital services. It also includes a question relating to time taken off work and/or education and usual activities due to experiencing a sore throat. These resource items are being documented by the participants over the one-week follow-up period and are being collected using a resource-use questionnaire/diary. In the questionnaire, participants log NHS services use: the number and type of GP or practice nurse visits (for example own home, clinic, practice, out-of-hours, phone), prescription use, over-the-counter medication use, and hospital Emergency Department, outpatient or inpatient stays that are directly related to their sore throat. This health service resource utilization will be valued using appropriate unit costs obtained from widely used sources, such as the most recent version of Unit Costs of Health and Social Care [22] and NHS reference costs.

EQ-5D-5L data is being collected using the standard instrument developed by the EuroQol group. The symptom and resource-use diary collects participant specific self-reported time away from work and/or education. Both are completed at baseline and over the seven day follow-up period.

Individual costs will be estimated by combining the resource use and unit cost data. We will estimate and report mean total costs by trial arm [23] and disaggregate these according to their burden on primary care and other care sectors. We will extrapolate our analysis of health service resource use and costs to explore the potential cost impact of prescribing oral corticosteroids on a national scale.

To aid decision-makers and to provide a transparency to our cost-effectiveness analysis we will analyze and report our costs and outcomes by trial allocation in a disaggregated format. Resource-use and costs will be reported by NHS sector. Outcomes will be reported in terms of symptom or pain-free days, EQ-5D-5L (overall scores and by domain) and days off work and/or education.

Mean costs and outcomes will be compared between the trial arms, using appropriate methods. The primary cost analysis will compare costs at one-month post-randomization. In the event of one treatment not dominating another, an incremental cost per quality-adjusted days (QAD) will be estimated using the EQ-5D-5L. Uncertainty in the confidence to be placed on the economic analysis results will be explored through deterministic and probabilistic sensitivity analysis and presented by estimating cost-effectiveness acceptability curves [24]. The sensitivity analyses will explore uncertainties in the trial data and analysis methods, including the possibility that consultation and re-consultation rates for those in the placebo arm may differ from current standard care.

#### The number of participants

Based on the results of our systematic review of eight studies, the average absolute increase in participants reporting complete resolution of pain at 24 hours with corticosteroids in addition to antibiotics and analgesia was 27% [15]. The minimum absolute increase from individual trials was 18% (11 versus 29%). To achieve this effect size with 90% power, our conservative estimate of sample size is 226 patients.

In the UK antibiotics are prescribed to approximately 50% of participants presenting with sore throat [1]. Given that our first secondary objective is to detect a clinically significant difference in proportions of participants not having been prescribed antibiotics, we will require an initial sample of 452 patients. A sample size of 566 allows for loss of up to 20% to follow-up (or 532 for 15% lost to follow-up).

#### Criteria for the termination of the trial

No formal interim analysis is planned to stop the trial early. Dexamethasone is already licensed and used at this dosage in a wide variety of disorders. In our systematic review we found no SAEs reported by any included trial. No differences were found in all AEs, relapse or recurrence rates between participants receiving corticosteroids and those receiving placebo, hence we anticipate that the likelihood of SAEs associated with a single dose of dexamethasone 10 mg taken orally will be extremely low. We have therefore not defined any criteria for termination for safety.

#### Procedure for accounting for missing, unused, and spurious data

The percentage of missing outcome data will be compared between two arms and a logistic model will be used to assess whether covariates significantly predict dropout. If little is known about the missing mechanism or there is any concern about validity of the expected missingness due to treatment failure (assuming no complete resolution), sensitivity analysis will be performed with plausible non-ignorable missing scenarios and complete cases. These will be detailed in the separate statistical analysis plan.

During statistical data review and analysis, any anomalies in the data will be investigated and discussed with the Trial Management Group. The data investigation will be broad and flexible and focus on variability of the data, consistency, dispersion, outliers, inliers, relationships between variables and relationships over time. The statistical data review will be fully documented with all the output dated.

#### Inclusion in analysis

We will be analyzing our data using the intention-to-treat principle. All eligible randomized participants will be included in the analysis, assuming no complete resolution for missing data.

### Direct access to source data and documents

Direct access is granted to authorized representatives from the Sponsor, host institution and the regulatory authorities to permit trial-related monitoring, audits and inspections. Individual GP practices are required to give access to the bodies described above and this is outlined in the Site Agreement.

### Quality control and quality assurance procedures

The study is being conducted in accordance with the current approved protocol, International Conference on Harmonization Good Clinical Practice, ICH GCP , relevant regulations and PC-CTU standard operating procedures. The monitoring will be performed by the PC-CTU Quality Assurance Manager or equivalent. All investigators and trial-related site staff have received training in trial procedures and ICH GCP.

Regular monitoring will be performed by the PC-CTU according to ICH GCP. Data is evaluated for compliance with the protocol and accuracy in relation to source documents. Following written standard operating procedures, the monitors will verify that the clinical trial is conducted and data are generated, documented and reported in compliance with the protocol, ICH GCP and the applicable regulatory requirements.

An independent Data Monitoring Committee, Trial Management Group and Trial Steering Committee have been appointed in line with standard clinical trials unit procedures. The responsibilities of the Data Monitoring Committee are to review and monitor the accruing data to ensure the rights, safety and wellbeing of the trial participants. They will provide an interim analysis if requested by the Trial Steering Committee. They will make recommendations to the Trial Steering Committee about how the study is operating, any ethical or safety issues and any data being produced from other relevant studies that might impact the trial. The Trial Management Group is responsible for the day to day running of the trial, including monitoring all aspects of the trial and ensuring that the protocol is being adhered to. The responsibilities of the Trial Steering Committee are to provide overall supervision of the trial on behalf of the Sponsor and the Funder to ensure that it is being conducted in accordance with ICH-GCP. The Trial Steering Committee will review the trial regularly, agree any amendments and provide advice on all aspects of the trial.

### Serious breaches

The Medicines for Human Use (Clinical Trials) Regulations, the UK legislation for the running of clinical trials, contain a requirement for the notification of ‘serious breaches’ to the MHRA within seven days of the Sponsor becoming aware of the breach. A serious breach is defined as: ‘A breach of GCP or the trial protocol which is likely to effect to a significant degree – (a) the safety or physical or mental integrity of the subjects of the trial; or (b) the scientific value of the trial’.

In the event that a serious breach is suspected the University of Oxford’s Sponsor’s office, CTRG, should be contacted within one working day of knowledge. Any possible serious breach is identified by a member of the study team; either through site monitoring or audit visits, or through a whistleblower or a complaint from within or outside the university. A written record of the incident will be made and once all necessary information is gathered the information is reviewed by the relevant staff (such as the Quality Assurance Manager) if appropriate and recorded on the Serious Breaches Assessment Form. If considered to be a serious breach the Chief Investigator will be asked to confirm this decision and to contact the CTRG. If the event is a serious breach the CTRG will inform the MHRA within seven days. Day 1 is considered as the day the incident is confirmed as serious by both the team and the CTRG. The incident will be followed up on by the CTRG in conjunction with the trials team. The PC-CTU will review all documentation to see what might have led to the breach and put in place a corrective action preventative action plan in collaboration with the CTRG.

### Ethics

The Chief Investigator ensures that this study is conducted in accordance with the principles of the Declaration of Helsinki. The Chief Investigator also ensures that this study is conducted in full conformity with relevant regulations and with the ICH Guidelines for Good Clinical Practice (CPMP/ICH/135/95) July 1996, including training in GCP for clinicians as required.

The protocol, informed consent form, participant information sheet and any proposed advertising material have received appropriate Research Ethics Committee (REC), regulatory authorities (MHRA in the UK), and host institution(s) approval (REC reference: 12/SC/0684 NRES Committee South Central - Oxford B). The Chief Investigator has submitted and will submit and, where necessary, obtain approval from the above parties for all substantial amendments to the original approved documents.

#### Participant confidentiality

The trial staff will at all times ensure that the participants’ anonymity is maintained. The participants are identified only by initials and a participant ID number on the CRF and any electronic databases. All documents are stored securely and only accessible by trial staff and authorized personnel. The study complies with the Data Protection Act, UK 1998, which requires data to be anonymized as soon as it is practical to do so.

#### Data handling and record keeping

Data management will be performed in accordance with PC-CTU standard operating procedure for data management. Study-specific procedures are outlined in a data management plan (DMP) to ensure that high-quality data are produced for statistical analysis. The DMP was reviewed and signed by all applicable parties including the Trial Manager and the Trial Statistician prior to the first patient being enrolled.

All patients are providing consent using pre-printed paper consent forms including the unique patient ID. Prepaid envelopes have been provided to return consent forms (and CRFs if applicable) to the trial centers, where the data is being entered by centre trial administrators.

Data collection and management is being conducted using a secure, web-based system developed in conjunction with the clinical trials unit. The system incorporates data entry and validation rules to reduce data entry errors, and management functions to facilitate auditing and data quality assurance. Parallel paper-based data capture forms will be available to those patients and clinicians who prefer this option. Data protection requirements are embedded into the design of the web-based system and enforced by best practice trial management procedures. The Clinical Data Manager will oversee the process of electronic data validation and manual listings, sending out data clarification forms (DCFs) when required and following these up until the queries are resolved.

Once the last patient is enrolled, prior to database lock a dataset review will be undertaken by the Information System Manager and Trial Statistician. All critical data items are 100% checked against original source data documents to ensure accuracy, and an error rate will be established across all fields to ensure a consistently accurate dataset.

Patient contact information is being collected at baseline in paper form and faxed to the relevant study centre. A copy of the patients contact details consent form will be sent to the PC-CTU. This information is being used to contact the patient to collect details for the primary outcome at 24, 48 and once more up to 96 hours after the patient has joined the trial, and for any further follow-up that might be required. The follow-up contact is being coordinated by a researcher at the relevant study centre. The contact details are being stored at the centre separately from all other trial data and will be anonymized as soon as the required contact has been completed.

At the conclusion of the trial and after the database has been locked, all essential documents will be archived for at least five years. The Chief Investigator is responsible for authorizing the retrieval and disposal of archived material.

#### Finance and insurance

The trial is funded by the National Institute for Health Research School for Primary Care Research, Project Number 172.

#### Compensation for harm

Indemnity and/or compensation for negligent harm arising specifically from an accidental injury for which the University is legally liable as the Research Sponsor will be covered by the University of Oxford. The NHS will owe a duty of care to those undergoing clinical treatment, with Trust Indemnity available through the NHS Litigation Authority Scheme. Indemnity and/or compensation for non-negligent harm (harm arising specifically from an accidental injury), and occurring as a consequence of the research subjects' participation in the trial for which the University is the Research Sponsor, will be covered by the University of Oxford.

### Publication policy

The investigators will be involved in reviewing drafts of the manuscripts, abstracts, press releases and any other publications arising from the study. Authors will acknowledge that the study was funded by the National Institute for Health Research, NIHR School for Primary Care Research. Authorship will be determined in accordance with the International Committee of Medical Journal Editors, ICMJE guidelines and other contributors will be acknowledged.

## Discussion

One of the challenges of designing a trial for an acute upper respiratory complaint is the fact that we are recruiting participants on the day that they present to their GP with a sore throat. There was concern that this might not leave participants sufficient time to fully understand the nature of the trial before consenting and receiving the medication. To counteract this we have developed very streamlined online recruitment procedures for the GPs, and have spent time with individual practices to ensure that the recruitment process can work within their usual care routine. These measures have ensured that participants are having enough time to read the participant information sheets outside of the actual clinic appointment so that when they see the recruiting clinician they will already have had time to process the information about the trial and can then simply ask any further questions they might have.

This trial is evaluating the effectiveness of a single oral corticosteroid dose in treating sore throat, but it will also help us to assess current antibiotic prescribing practices in this situation. We will be able to see whether delayed antibiotic prescriptions are being given out, whether these are being used once given and whether people are simply re-presenting to primary care or out-of-hours if they are not given an antibiotic prescription.

## Trial status

The trial is currently open and recruiting in all centers and currently on target. We are aiming to complete recruitment by December 2014 and patient follow-up by February 2015.

## Electronic supplementary material

Additional file 1: **Study flow chart.** (DOC 70 KB)

Additional file 2: **Schedule of procedures.** (DOC 55 KB)

## Abbreviations

AE: Adverse event
AR: Adverse reaction
CRF: Case Report Form
CRO: Contract Research Organization
CT: Clinical Trials
CTA: Clinical Trials Authorization
CTRG: Clinical Trials & Research Governance University of Oxford
DMC: Data Monitoring Committee
GCP: Good Clinical Practice
GP: General Practitioner
IB: Investigators Brochure
ICF: Informed Consent Form
ICH: International Conference of Harmonization
ICMJE: International Committee of Medical Journal Editors
IMP: Investigational Medicinal Product
IRB: Independent Review Board
MHRA: Medicines and Healthcare products Regulatory Agency
NRES: National Research Ethics Service
PI: Principal Investigator
PIL: Participant/ Patient Information Leaflet
R&D: NHS Trust R&D Department
REC: Research Ethics Committee
SAE: Serious Adverse Event
SAR: Serious Adverse Reaction
SmPC: Summary of Product Characteristics
SUSAR: Suspected Unexpected Serious Adverse Reactions
TMF: Trial Master File
TMG: Oxford Radcliffe Hospitals Trust / University of Oxford Trial Management Group
TSC: Trial Steering Committee.
