# Supplementary material for: Oral corticosteroid use for clinical and cost-effective symptom relief of sore throat: study protocol for a randomized controlled trial
Source: Trials. 2014 Sep 18;15:365. doi: 10.1186/1745-6215-15-365 (PMC4182852; doi:10.1186/1745-6215-15-365)
Supplement: Supplementary file 1 — Additional file 1: Study flow chart. (DOC 70 KB) [file 13063_2013_2240_MOESM1_ESM.doc]

# APPENDIX A: STUDY FLOW CHART

Allocated to single dose of oral 10mg (5 x 2mg tablets, over encapsulated) dexamethasone

Allocated to single dose of placebo (5 tablets, over encapsulated) with identical packaging

Completion of online or paper diary over 7 days including

Complete resolution of symptoms

Symptom duration

Use of delayed antibiotic prescription

Cost effectiveness data

Notes review at one month to establish further use of health care resources, prescription medications, adverse events and complications.

Estimated loss to follow-up of (n = 114)

Receive delayed antibiotic prescription

(predicted n = 283)

Receive no delayed antibiotic prescription

(predicted n = 283)

Patient’s ≥ 18 years old presenting with less than one week duration of:

complaint of sore throat

odynophagia

Excluded

Exclusion criteria met

Declined to participate

Screening:

Eligibility assessed with reference to exclusion criteria.

Patient Information Sheet provided, study explained by general practitioner or research or practice nurse.

Delayed antibiotic prescription offered according to clinical judgement, to be collected at Baseline Trial Assessment.

Baseline Trial Assessment:

Written informed consent obtained.

Randomised according to stratification below
