# Supplementary material for: Oral corticosteroid use for clinical and cost-effective symptom relief of sore throat: study protocol for a randomized controlled trial
Source: Trials. 2014 Sep 18;15:365. doi: 10.1186/1745-6215-15-365 (PMC4182852; doi:10.1186/1745-6215-15-365)
Supplement: Supplementary file 2 — Additional file 2: Schedule of procedures. (DOC 55 KB) [file 13063_2013_2240_MOESM2_ESM.doc]

# APPENDIX B: SCHEDULE OF PROCEDURES

| **30 Day follow up** |  |  | 🗸 | 🗸 |  |  |  |  |  |  |  |  | 🗸 |  |  |  | 🗸 | ^1^ Performed by GP Practice staff- Responsible / Recruiting Clinician  ^2^ Performed by GP  ^3^ Performed by local centre  ^4^ Performed by Patient  ^5^ If Needed  ^6^ Either/ Or |
| --- | --- | --- | --- | --- | --- | --- | --- | --- | --- | --- | --- | --- | --- | --- | --- | --- | --- | --- |
| **Day 14** |  |  |  |  |  |  |  |  |  |  |  |  |  |  |  | 🗸^5^ |  |
| **Day 7** |  |  |  |  |  |  |  |  |  |  |  |  |  |  | 🗸 |  |  |
| **Day 6** |  |  |  |  |  |  |  |  |  |  |  |  |  |  | 🗸 |  |  |
| **Day 5** |  |  |  |  |  |  |  |  |  |  |  |  |  |  | 🗸 |  |  |
| **Day 4** |  |  |  |  |  |  |  |  |  |  |  |  |  | 🗸^5^ | 🗸 |  |  |
| **Day 3** |  |  |  |  |  |  |  |  |  |  |  |  |  |  | 🗸 |  |  |
| **Day 2** |  |  |  |  |  |  |  |  |  |  |  |  |  | 🗸 | 🗸 |  |  |
| **Day 1** |  |  |  |  |  |  |  |  |  |  |  |  |  | 🗸 | 🗸 |  |  |
| **Baseline** | 🗸^6^ | 🗸 | 🗸 | 🗸 | 🗸 | 🗸 |  | 🗸 | 🗸 | 🗸 |  | 🗸 |  |  | 🗸 |  |  |
| **Screening** | 🗸^6^ |  |  |  |  |  | 🗸 |  |  |  | 🗸 |  |  |  |  |  |  |
| **Actions** | **Informed consent^1^** | **Demographics^1^** | **Medical history^1^** | **Concomitant medications^1^** | **Physical examination^1^** | **Throat Swab^1^** | **Eligibility assessment^1^** | **Randomisation^1^** | **Dispensing of study drugs^1^** | **Compliance^1^** | **Writing of Prescription^2^** | **Giving of Prescription^1^** | **Adverse event assessments^2^** | **Follow-up Contact^3^** | **Symptom Diary^4^** | **Follow-up Questionnaire^4^** | **Review of NHS usage** |
